# Characterization and expression analysis of the SPL gene family during floral development and abiotic stress in pecan (*Carya illinoinensis*)

**DOI:** 10.7717/peerj.12490

**Published:** 2021-12-09

**Authors:** Min Wang, Zhenghai Mo, Ruozhu Lin, Cancan Zhu

**Affiliations:** 1 Institute of Botany, Jiangsu Province and Chinese Academy of Sciences, Nanjing, China; 2 Horticulture Research Institute, Shanghai Key Lab of Protected Horticultural Technology, Shanghai Academy of Agricultural Sciences, Shanghai, China; 3 Key laboratory of Forest Protection of National Forestry and Grassland Administration, Research Institute of Forest Ecology, Environment and Protection, Chinese Academy of Forestry, Beijing, China

**Keywords:** *Carya illinoinensis*, SPL gene, Phylogenetic analysis, Gene promoter, Flower development, Abiotic stress

## Abstract

SQUAMOSA promoter binding protein-like (*SPL*) genes are a type of plant-specific transcription factors that play crucial roles in the regulation of phase transition, floral transformation, fruit development, and various stresses. Although *SPLs* have been characterized in several model species, no systematic analysis has been studied in pecans, an important woody oil tree species. In this study, a total of 32 *SPL* genes (*CiSPLs*) were identified in the pecan genome. After conducting phylogenetic analysis of the conserved SBP proteins from *Arabidopsis*, rice, and poplar, the *CiSPLs* were separated into eight subgroups. The *CiSPL* genes within the same subgroup contained very similar exon-intron structures and conserved motifs. Nine segmentally duplicated gene pairs in the pecan genome and 16 collinear gene pairs between the *CiSPL* and *AtSPL* genes were identified. Cis-element analysis showed that *CiSPL* genes may regulate plant meristem differentiation and seed development, participate in various biological processes, and respond to plant hormones and environmental stresses. Therefore, we focused our study on the expression profiles of *CiSPL* genes during flower and fruit development. Most of the *CiSPL* genes were predominantly expressed in buds and/or female flowers. Additionally, quantitative real time PCR (qRT-PCR) analyses confirmed that *CiSPL* genes showed distinct spatiotemporal expression patterns in response to drought and salt treatments. The study provides foundation for the further exploration of the function and evolution of *SPL* genes in pecan.

## Introduction

SQUAMOSA promoter binding protein-like (*SPL)* genes make up a unique transcription factor family that widely exists in green plants and plays a key role during floral development and stress response. Two *SPLs* originally isolated from snapdragon (*Antirrhinum majus*) were named *SBP1* and *SBP2* (Klein, Saedler & Huijser, 1996). With the recent completion of the whole genome, many members of the SPL gene family have been identified in various species such as rice, *Arabidopsis*, Chinese cabbage, tomato, soybean, citrus, apple, grape, jujube, and chestnut (Yang et al., 2008; Salinas et al., 2012; Tripathi et al., 2017; Hou et al., 2013; Tan et al., 2015; Shalom et al., 2015; Shao et al., 2017; Li et al., 2013; Chen et al., 2019). Each *SPL* gene contains a highly conserved SBP domain that is usually the size of about 78 amino acids and includes a nuclear localization signal and two zinc fingers (Yamasaki et al., 2004). SPL members function not only as transcription factors, but also as miR156/157 targets that form a complex gene regulatory network (Jia et al., 2018).

*SPLs* are involved in plant development processes, including shoot and leaf morphogenesis, floral organ development, flowering, and fruit ripening (Preston & Hileman, 2013; Xu et al., 2016; Yu et al., 2015; Wang et al., 2016; Gou et al., 2011). In *Arabidopsis*, six *SPLs* (*SPL2*, -*9*, -*10*, -*11*, -*13*, and -*15*) regulate various processes during shoot and root development (Wu et al., 2009; Wang et al., 2008; Stief et al., 2014; Yu et al., 2010; Usami et al., 2009; Yu et al., 2015). However, *SPL3*, *-4*, and *-5* may promote floral meristem identity (Wu & Poethig, 2006; Yamaguchi et al., 2009; Wang, Czech & Weigel, 2009; Cardon et al., 1997) and Arabidopsis *SPLs* directly activate flower meristem specific genes (*LFY*, *FUL*, and *AP1*) to promote the transition from vegetative to reproductive growth (Yamaguchi et al., 2009). Similar to *Arabidopsis*, the single *AtSPL3/4/5* ortholog *AmSBP1* in snapdragon (*Antirrhinum majus*) was found to be involved in the initiation of flower development after the switch to inflorescence development (Klein, Saedler & Huijser, 1996).

*SPL* genes are also involved in fruit development. Studies have shown that *AtSPL8* affects the development of pollen sacs (Unte et al., 2003). In rice, it has been found that *OsSPL13* and *OsSPL16* regulate grain size and shape (Si et al., 2016; Wang et al., 2012a; Wang et al., 2012b). *OsSPL13* overexpression in rice not only increases the rice grain size, but also the spike length, the number of grains per spike (significantly), and the ultimate rice yield. *OsSPL16* gene overexpression promotes endosperm cell proliferation and grain filling, and increases grain width, grain weight, and yield.

Additionally, *SPL* genes play important roles in abiotic stress response. *AtSPL1* and *AtSPL12* participate in the regulation of thermotolerance during reproductive growth in *Arabidopsis thaliana,* and *AtSPL1* and *AtSPL2* overexpression enhances inflorescence thermotolerance (Chao et al., 2017). *OsSPL10* plays dual roles in trichome formation and salt tolerance in rice: it has a negative effect on salt stress and a positive effect on the formation of trichomes (Lan et al., 2019). *MsSPL8* gene down-regulation improves drought and salt tolerance in transgenic alfalfa (Gou et al., 2018). Birch *SPL9* (*BpSPL9*) was expressed in roots and leaves under NaCl and PEG6000 stress (Ning et al., 2017). Physiological and enzymological analyses of *BpSPL9* transgenic lines in birch showed that ROS scavenging improved under salt and drought stress and *BpSPL9* gene overexpression enhanced salt and drought tolerance (Ning et al., 2017). The Chinese wild Vitis species *SBP-box* gene (*VpSBP16*) showed enhanced tolerance to drought and salt stress in transgenic *Arabidopsis* by regulating ROS and SOS signal networks (Hou et al., 2018). Some *ZmSPL* genes were induced by various environmental stimuli, such as cold, drought, and salinity (Mao et al., 2016).

Pecan (*Carya illinoinensis*), a hickory of the walnut family, is an important woody oil tree species. The oil content in its seed kernel is more than 70%, with unsaturated fatty acids making up 97% of that amount (Huang et al., 2017). The market demand for pecans is steadily increasing, indicating that the pecan industry still has a huge market prospect. However, pecan has a long vegetative growth period, meaning late flowering and low yields, which has seriously restricted the development of its industry. In order to improve the yield and benefit of pecan, it is necessary to regulate the balance between vegetative and reproductive growth, initiate the expression of flowering factors, and promote flower bud differentiation. It is well known that *SPL* genes regulate inflorescence and fruit development along with signal transduction and physiological and biochemical processes. Therefore, studying the function of *CiSPL* genes is of great significance in research on the flowering mechanism of pecan. In our study, we identified 32 *CiSPL* genes from pecan and performed a comprehensive analysis of its phylogenetic relationships, collinearity, gene structure, conserved motif, and cis-acting regulatory elements. The expression patterns of 32 *CiSPL* genes at different stages of female flower bud differentiation and fruit development, as well as in response to drought and salt stress treatment, were detected by qRT-PCR. Our research provides a comprehensive analysis of the CiSPL gene family and will be helpful in the further study of pecan *SPL* gene evolution and function.

## Materials & Methods

### Identification and characterization of SPL genes in pecan

The genome database, protein sequence, and pecan CDS sequences were downloaded from the Hardwood Genomics Project (https://www.hardwoodgenomics.org/) (Huang et al., 2019). The protein sequences of *Arabidopsis*, rice, and poplar SPL gene families were downloaded from the Plant Transcription Factor Database (PlantTFDB). The protein domain sequences of SPLs from the other three species were used as query sequences to blast the pecan proteome data with Local Blast P retrieval, and the *E* value was set to 1e−6. The blast results were sorted out to remove the repetitive sequence, and the NCBI-CDD (https://www.ncbi.nlm.nih.gov/cdd) and online software Pfam (http://pfam.xfam.org/) were used to further verify. The physical and chemical properties were analyzed using the online software Expasy (https://web.expasy.org/protparam/), and subcellular localization was predicted using Wolf psort (https://www.genscript.com/tools/wolf-psort).

### Classification and phylogenetic tree construction of the SPL gene family

Bioedit software and the conserved domain sequences of SBP proteins in pecan were used to carry out multiple sequence alignments. For the evolutionary relationship, the candidate SPL protein domains of pecan, rice, *Arabidopsis*, and poplar were analyzed for multiple sequence alignment using ClustalW. Then, the comparison results were analyzed using MEGA 5.0 with a maximum likelihood (ML) (bootstrap=500) to construct the phylogenetic tree. Another phylogenetic tree was constructed using the SPL domains of all pecan protein sequences for further analysis via the same method.

### Gene duplication and Ka/Ks value calculations

The collinearity (synteny) in the pecan, and between pecan and *Arabidopsis,* were analyzed using MCScanX software, and the *E*-value was set as 1  × 10^−5^, in accordance with previous studies (Wang et al., 2012). The ratio of nonsynonymous to synonymous nucleotide substitutions (Ka/Ks) was evaluated among segmentally duplicated gene pairs to detect the selection mode. First, the nucleotide coding sequences (CDS) of the *CiSPL* genes were aligned using the Muscle program with default options in MEGA 5.0 according to the protein sequence alignments. The Ka/Ks was then calculated based on multiple sequence alignments using MEGA 5.0 (Hu et al., 2019).

### SPL gene structure and conserved motif analysis

GSDS software was employed to analyze gene structure. Simultaneously, we used the online software MEME (http://meme-suite.org/tools/meme) to predict and analyze the protein conservative motif of the SPL protein sequences. The MEME parameters were set as follows: maximum number of motifs, 20; motifs width: 6-60.

### Category and number of cis-acting elements in *CiSPL* promoters

We extracted 1,500 bp promoter regions upstream of the translation start codon (ATG) of the *CiSPL* genes from the pecan genome website, which we labeled as promoter fragments. The cis-acting elements of each *CiSPL* were identified using the online program PlantCARE (Lescot et al., 2002).

### Expression profiles of *CiSPLs* at various stages of flower and fruit development

In order to explore the role of *CiSPLs* in pecan growth and development, a comprehensive expression profile analysis was carried out. Normalized expression levels during female flower development were retrieved from our previous RNA-Seq data (Wang et al., 2019), and the *CiSPL* gene expression data in fruit development was retrieved from RNA transcriptome data (Bioproject ID PRJNA435846). The calculated fragments per kb of exon per million mapped reads (FPKM) were used to normalize the gene expression values (Huang et al., 2019). Next, the log2(FPKM) values of the *CiSPL* genes were used to generate heat maps with the software MEV4.9.

### Plant materials and stress treatment

The gene expression patterns of 32 *CiSPLs* were detected in the leaves of pecan seedlings at the Institute of Botany, Jiangsu province and the Chinese Academy of Sciences. The pecan seedlings were cultivated from the seeds with the same size of ‘Pawnee’ and grown in fields under natural conditions. At the four-true-leaf stage, uniform and healthy pecan seedlings were transferred into a greenhouse. After being cultured in Hoagland’s solution for seven days, pecan seedlings were subjected to a variety of abiotic stresses. We added 10% polyethylene glycol (PEG) 6000 to the solution for drought treatment, and 3‰ (w/v) NaCl to the nutrient solution for salt treatment (Mo et al., 2020). Young leaves from the stress-treated plants were collected 0, 6, 12, and 24 h after treatment. All samples were immediately frozen in liquid nitrogen and stored at −80 °C until RNA extraction. The untreated pecan seedlings were used as the control groups.

### RNA extraction and real time PCR analysis

Total RNA was isolated using the Universal Plant RNA Extraction Kit (BioTeke, Beijing, China), and reverse transcribed to cDNA with the PrimeScript™ RT reagent Kit (TaKaRa, Kyoto, Japan). The gene-specific primers of 32 *CiSPLs* were designed using CDS sequences and Primer 5.0 software, and all the primers are listed in Table S1. qRT-PCR was performed on ABI StepOnePlus (Thermo Fisher Scientific, Waltham, MA, USA) with SYBR Green Realtime PCR Master Mix (Thermo Fisher). Each reaction volume was 20 µL, and included 1 µL of diluted cDNA template, 0.4 µL of each gene forward and reverse primer, 10 µL of SYBR Mix, 8.2 µL of ddH2O, and 0.4 µL of ROX Reference Dye. The PCR reaction procedure was as follows: 95 °C for 30s, followed by 40 cycles at 95 °C for 5 s, 60 °C for 34 s, and the dissolution curve reaction procedure (95 °C for 15 s, 60 °C for 1 min, and 95 °C for 15 s). The pecan *actin* gene was used as the internal reference gene (Zhang et al., 2016). The relative expression level for all *CiSPL* genes was calculated using the 2^−ΔΔCt^ method (Livak & Schmittgen, 2001). ANOVA Duncan analysis was performed for significant difference analysis using SPSS 21.0 software.

## Results

### Identification and annotation of *SPLs* in pecan

Referencing the SPL protein sequences of Arabidopsis, rice, and poplar, we searched all assumptive *SPL* genes of pecan using Local BLAST and BioEdit software. Through Pfam and NCBI domain analysis, we identified 32 candidate *SPL* genes (Table 1). Previously, Wu et al. (2019) found 30 *CiSPL* genes in the pecan genome. However, in this study, we found two *CiSPL* genes (*CiSPL4c*, *CIL1118S0019*; *CiSPL9d*, *CIL1312S0023*) in addition to the previously identified 30 *CiSPL* genes. The SBP box domains of the 32 CiSPL proteins were aligned and analyzed using BioEdit software (Table. S1). All *CiSPLs* had two zinc finger-like structures and NLS fragments except for *CiSPL4c*, *CiSPL7b*, and *CiSPL9d*, which indicated that the SBP domain was conserved in pecan. Further, the *CiSPLs* were named by orthology with *Arabidopsis SPLs*. The characterizations of CiSPL proteins were analyzed using the ProtParam tool. The lengths of the 32 CiSPL proteins ranged from 108 (*CiSPL4c*) to 1,071 (*CiSPL16a*), with molecular weights ranging from 12.184 kDa to 118.955 kDa, and theoretical isoelectric points from 5.25 to 9.36. This range of variations means that different CiSPL proteins may operate in different microenvironments. Most *CiSPL* genes were located in the nucleus, only two genes (*CiSPL4c* and *CiSPL7b)* were located in the chloroplast.

**Table 1:** Identification, classification and physicochemical properties of *CiSPL* genes.

Name	Gene Id	Gene location	Group	CDS length (bp)	Size (aa)	Protein MW (KDa)	PI	Loc
*CiSPL2a*	CIL1138S0014	scaffold87792: 121998: 128864.+	V	1041	346	38.216	8.73	nucl
*CiSPL2b*	CIL1486S0026	scaffold122014: 339920: 354017.+	V	1398	465	51.780	8.28	nucl
*CiSPL3a*	CIL0893S0386	scaffold68792: 4195186: 4201886.-	VI	444	147	16.669	7.05	nucl
*CiSPL3b*	CIL1086S0071	scaffold82459: 946930: 952277.-	VI	456	151	17.445	7.66	nucl
*CiSPL4a*	CIL1078S0073	scaffold81904: 533329: 534617.+	VI	597	198	22.092	9.19	nucl
*CiSPL4b*	CIL1361S0046	scaffold109903: 544411: 546249.+	VI	621	206	22.945	9.12	nucl
*CiSPL4c*	CIL1118S0019	scaffold85792: 413380: 413706.-	VI	327	108	12.184	8.96	chlo
*CiSPL5*	CIL0909S0046	scaffold70015: 471192: 473177.-	VI	666	221	25.254	5.25	nucl
*CiSPL6a*	CIL1066S0023	scaffold81126: 457953: 462330. +	IV	1596	531	58.830	7.07	nucl
*CiSPL6b*	CIL1182S0013	scaffold93126: 195544: 201022.+	IV	1584	527	58.235	7.88	nucl
*CiSPL6c*	CIL1032S0023	scaffold77681: 289083: 314501.+	IV	1629	542	59.410	6.81	nucl
*CiSPL6d*	CIL0248S0001	scaffold22449: 2215: 12505.-	IV	1683	560	62.434	6.33	nucl
*CiSPL7a*	CIL1369S0030	scaffold110459: 462859: 474068.-	I	2385	794	89.169	6.90	nucl
*CiSPL7b*	CIL0037S0017	scaffold3226: 140675: 158469.+	I	1932	643	71.923	8.30	chlo
*CiSPL8a*	CIL1479S0028	scaffold121347: 434913: 437667.-	III	942	313	35.019	8.99	nucl
*CiSPL8b*	CIL1609S0008	scaffold134903: 248011: 250331.-	III	1089	362	40.668	9.17	nucl
*CiSPL9a*	CIL1047S0099	scaffold79681: 1350803: 1355813.+	VIII	1146	381	40.575	9.20	nucl
*CiSPL9b*	CIL0987S0054	scaffold74570: 467811: 471556.+	VIII	1134	377	40.316	9.36	nucl
*CiSPL9c*	CIL1479S0027	scaffold121347: 418158: 424186.+	VIII	1194	397	43.682	6.85	nucl
*CiSPL9d*	CIL1312S0023	scaffold104459: 174377: 175048.-	VIII	672	223	24.786	7.67	nucl
*CiSPL12a*	CIL0118S0017	scaffold10448: 265972: 274738.-	II	3012	1003	111.793	5.58	nucl
*CiSPL12b*	CIL1028S0077	scaffold77237: 1437361: 1446283.-	II	3024	1007	111.896	6.24	nucl
*CiSPL12c*	CIL0317S0003	scaffold31337: 12553: 21322.+	II	3111	1036	115.511	7.02	nucl
*CiSPL12d*	CIL1198S0043	scaffold95015: 592578: 602173.+	II	2973	990	109.897	6.86	nucl
*CiSPL13a*	CIL1101S0006	scaffold84015: 175449: 179291.-	VII	1179	392	43.110	6.73	nucl
*CiSPL13b*	CIL1064S0113	scaffold81015: 1301406: 1305416.-	VII	1170	389	42.978	8.73	nucl
*CiSPL13c*	CIL0984S0053	scaffold74459: 902792: 906703.-	VII	963	320	34.966	9.09	nucl
*CiSPL13d*	CIL1086S0008	scaffold82459: 85681: 87820.+	VII	972	323	35.399	8.93	nucl
*CiSPL13e*	CIL1145S0047	scaffold88681: 453966: 456433.+	VII	1149	382	41.903	7.99	nucl
*CiSPL13f*	CIL1515S0030	scaffold124569: 381271: 383997.-	VII	1173	390	43.411	6.07	nucl
*CiSPL16a*	CIL1230S0089	scaffold97903: 847870: 854123.-	II	3216	1071	118.955	8.24	nucl
*CiSPL16b*	CIL1294S0032	scaffold103237: 455137: 460718.+	II	3204	1067	118.346	7.70	nucl

**Notes.**

AA amino acid residues MW molecular weight pI theoretical isoelectric point Loc subcellular location Nucl nucleus Chlo chloroplast

### Cluster analysis and phylogenetic tree construction of SPL proteins in pecan

**Figure 1:**
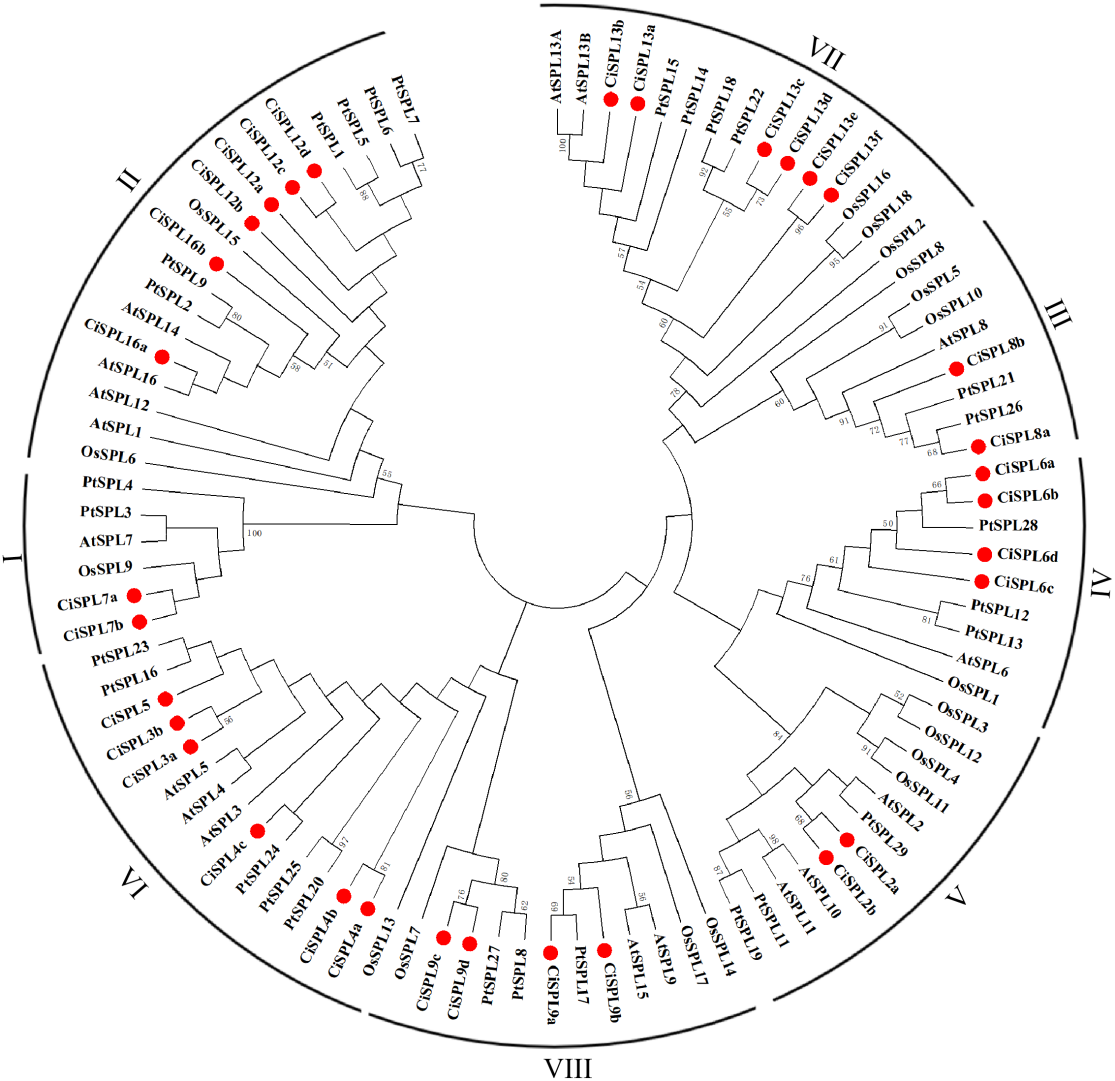
Phylogenetic analysis of the SPL family in pecan, *Arabidopsis*, rice, and poplar. Phylogenetic tree of conserved SPL domains from pecan (CiSPL), *Arabidopsis* (AtSPL), rice (OsSPL), and poplar (PtSPL). The tree was constructed using MEGA 5.0 software with the Maximum Likelihood (ML) method, and bootstrap values were calculated with 500 replicates. The subgroups are shown outside of the tree. Bootstrap values greater than 50% are displayed.

**Figure 2:**
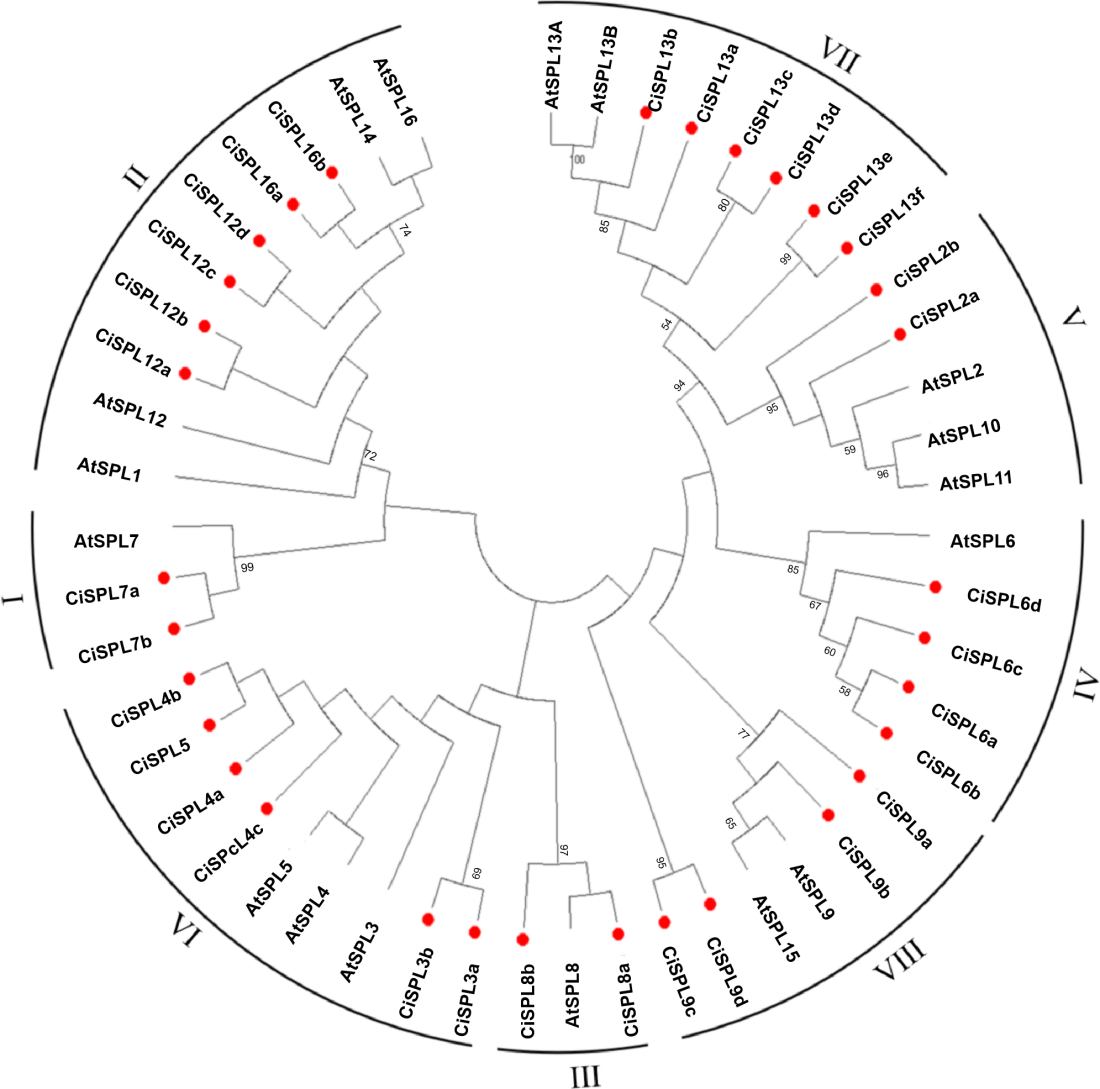
Phylogeny and distribution of SPL proteins from pecan and *Arabidopsis*. The tree phylogenetic was generated using the Maximum Likelihood (ML) method with 500 bootstrap replicates by MEGA 5.0 software. Bootstrap values greater than 50% are displayed.

In order to explore the evolutionary relationships of SPL proteins in pecan and other species, we used the ML method on the SBP domains of 95 SPL proteins from pecan (32), *Arabidopsis* (17), rice (18), and poplar (28) to construct the phylogenetic tree (Fig. 1). In order to better understand the phylogenetic relationship of the SPL members, we also performed multiple alignment and phylogenetic tree analyses on the core SBP domains of all CiSPLs and AtSPLs. The 32 *CiSPL* genes were divided into eight subgroups (from I to VIII), described previously, based on phylogenetic analysis and SBP domain alignment (Fig. 2). The subgroups II, VI, and VII had the largest number of members (six). Each subgroup contained at least one *SPL* gene from the other three species (*Arabidopsis*, rice, and poplar). Subgroup I contained one *SPL* gene from Arabidopsis and rice, and two from pecan and poplar, suggesting the functional conservation of the *SPL7* gene in plants. *CiSPL* genes showed high similarity with the poplar orthologs, and had the most similar number of genes in each subgroup. The SBP domain was relatively conservative in different species.

### Motif and gene structure analysis of *SPL* genes in pecan

**Figure 3:**
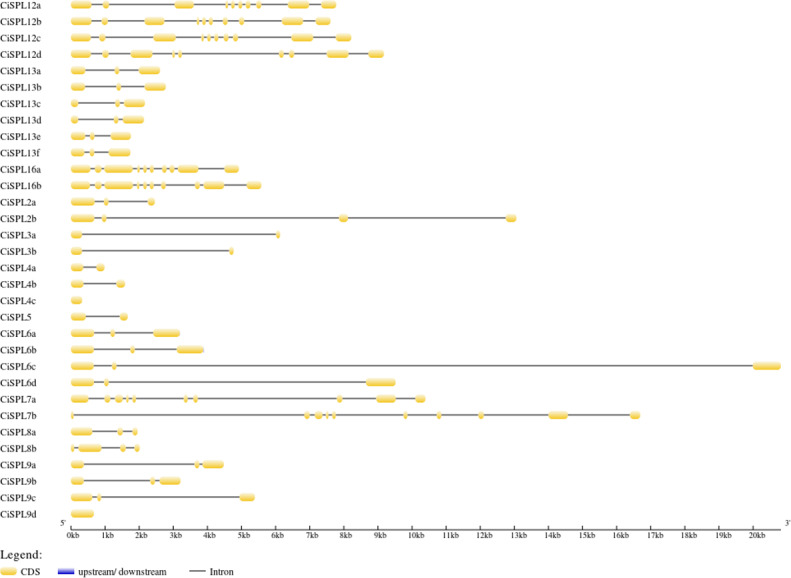
The gene structure of *CiSPL* genes. The exons and introns are indicated by yellow blocks and gray thin lines, respectively. 5′UTR and 3′UTR are not shown.

The *CiSPL* gene structure was analyzed using GSDS 2.0 (http://gsds.cbi.pku.edu.cn/) (Fig. 3). The number of introns in the 32 *CiSPLs* ranged from 0 to nine. Almost half of the 32 SPL genes (15 *CiSPLs*) had two introns. There were nine introns in seven *CiSPLs* (mainly members of group I and II), *CiSPL2b* and *CiSPL8b* had three introns each, *CiSPL4c* and *CiSPL9d* had no introns, and the remaining six *CiSPL* genes contained only one intron each. Most of the *CiSPL* genes within the same subgroups showed similar gene structures in terms of intron number and exon length. *CiSPL3a* and *CiSPL3b* were in group VI, and the length of their protein sequences was shorter than 200 amino acids, while in subgroup II, the SPL protein sequence was longer than 1,000 amino acids.

**Figure 4:**
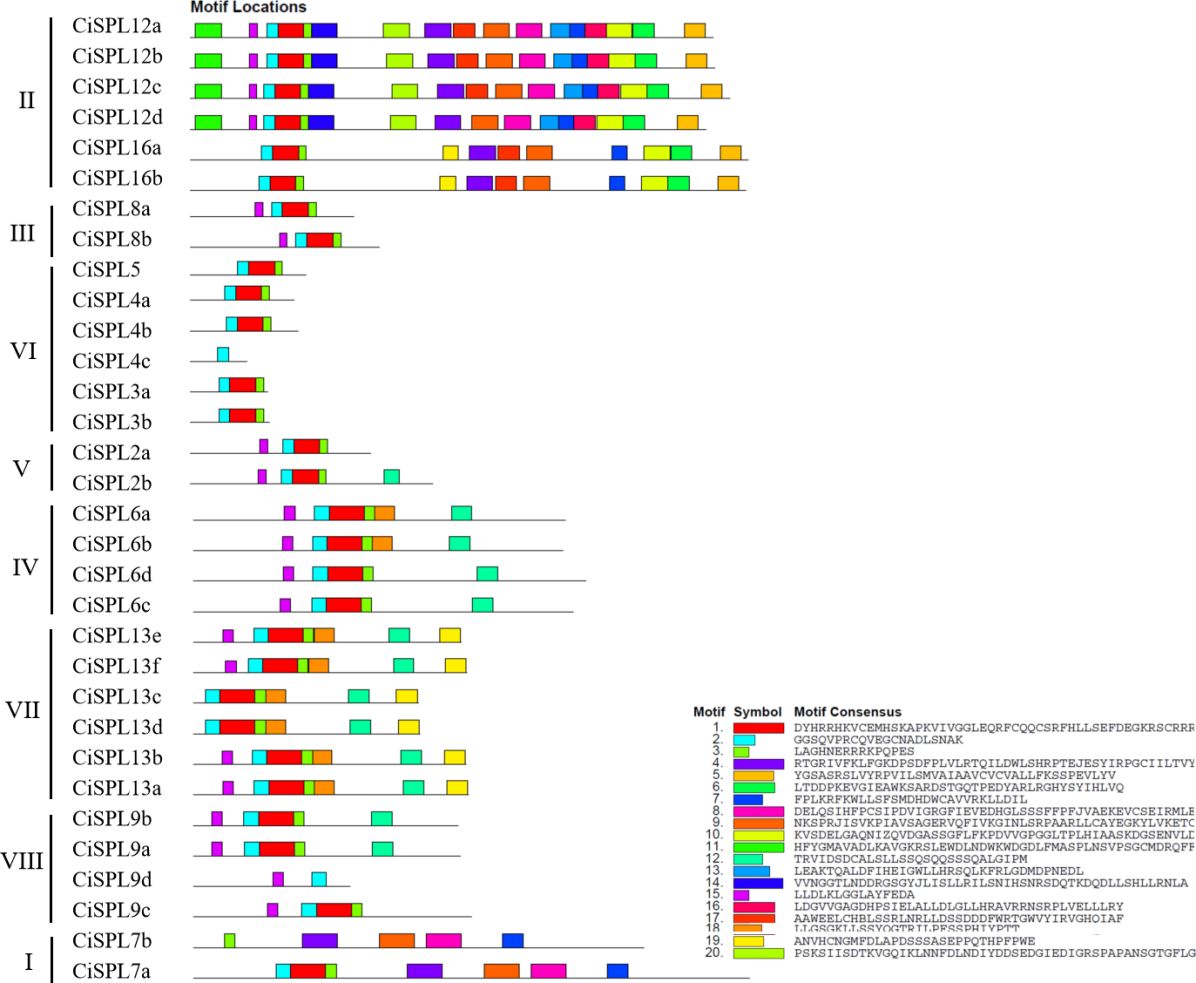
The conserved motifs distributed in CiSPL proteins were predicted by MEME. Different motifs are represented by different colors and numbered from 1 to 20. The legend includes color definitions.

Other conserved domains besides the SBP domain also played roles in protein function. Based on the MEME software analysis of 32 CiSPL protein sequences, 20 motifs were identified and numbered from 1 to 20 (Fig. 4, Table S2). The number of motifs ranged from one to 17 in each SPL protein. All CiSPL proteins (except for CiSPL4c, CiSPL7b, and CiSPL9d) contained motif 1 (Zn-2), motif 2 (Zn-1), and motif 3 (NLS). CiSPL7b contained only motif 3, while CiSPL4c and CiSPL9d only had motif 2. Five CiSPL proteins (CiSPL3a, CiSPL3b, CiSPL4a, CiSPL4b, and CiSPL5) contained only motif 1, motif 2, and motif 3, while other CiSPL proteins had more motifs. CiSPL12a, CiSPL12b, CiSPL12c, and CiSPL12d had 17 motifs, and CiSPL16a and CiSPL16b had 11 motifs. Members of the same clade showed similar gene structures.

**Table 2:** The Ka/Ks values of the collinear gene pairs of *SPLs* in pecan and *Arabidopsis*.

Gene pairs	Ka/Ks
CiSPL2a/CiSPL2b	0.38
CiSPL3a/CiSPL5	0.37
CiSPL4a/CiSPL4b	0.27
CiSPL6a/CiSPL6b	0.37
CiSPL7a/CiSPL7b	0.41
CiSPL9a/CiSPL9b	0.34
CiSPL12a/CiSPL12b	0.30
CiSPL13e/CiSPL13f	0.43
CiSPL16a/CiSPL16b	0.24
CiSPL3a/AtSPL3	0.13
CiSPL3b/AtSPL3	0.11
CiSPL4b/AtSPL5	0.33
CiSPL6a/AtSPL6	0.37
CiSPL6b/AtSPL6	0.39
CiSPL7a/AtSPL7	0.32
CiSPL7b/AtSPL7	0.33
CiSPL9a/AtSPL9	0.32
CiSPL9a/AtSPL15	0.28
CiSPL9b/AtSPL9	0.28
CiSPL9b/AtSPL15	0.32
CiSPL12b/AtSPL1	0.18
CiSPL13b/AtSPL13A	0.36
CiSPL16a/AtSPL14	0.19
CiSPL16a/AtSPL16	0.16
CiSPL16b/AtSPL16	0.18

### Gene duplication and collinearity analysis of *CiSPL* genes

**Figure 5:**
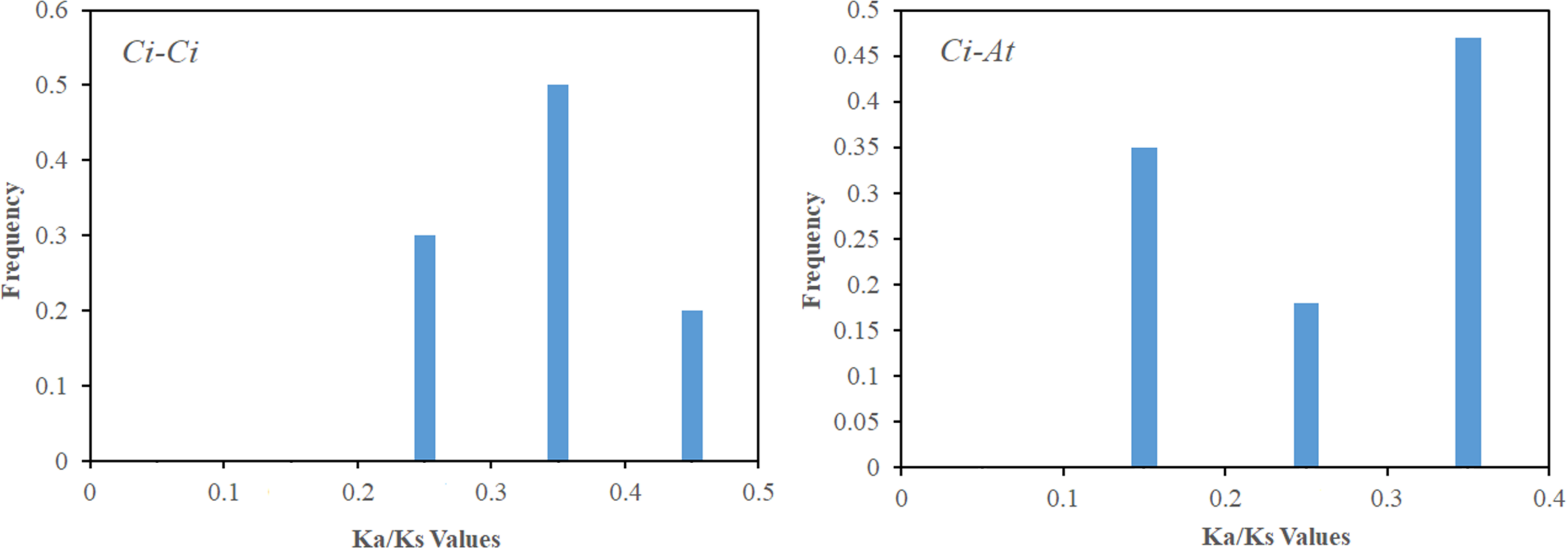
The Ka/Ks value distribution of the segmental duplication gene pairs in the pecan genome , and the collinear gene pairs between pecan and *Arabidopsis*. Distribution of Ka/Ks values was obtained from segmentally duplicated gene pairs in the pecan genome (A), and the collinear gene-pairs between pecan and *Arabidopsis* (B).

Gene duplication is both the foundation of gene function diversification and the main driving force of species evolution. In order to explore the expansion and evolutionary mechanism of the CiSPL gene family, MCScanX software was used to analyze the *SPL* gene replication mode. Twelve genes were involved in nine segmental duplication events (Table 2). However, there were no tandem duplication events, which suggests that segmental duplications were the main mechanism of pecan SPL gene expansion. In order to further understand the evolutionary and functional relationship between the pecan and Arabidopsis SPL genes, we analyzed their collinearity relationships. The 16 collinear gene pairs, which included 13 *CiSPLs* and 11 *AtSPLs*, were as follows: *CiSPL3a/AtSPL3*, *CiSPL4b/AtSPL5, CiSPL6a/AtSPL6*, *CiSPL6b/AtSPL6*, *CiSPL7a/AtSPL7, CiSPL7b/AtSPL7*, *CiSPL9a/AtSPL9*, *CiSPL9a/AtSPL15*, *CiSPL9b/AtSPL9*, *CiSPL9b/AtSPL15*, *CiSPL12b/AtSPL1*, *CiSPL13b/AtSPL13A*, *CiSPL3b/AtSPL3*, *CiSPL16a/AtSPL14*, *CiSPL16a/AtSPL16, and CiSPL16b/AtSPL16*. This suggested that most *SPLs* had orthologs in Arabidopsis. For each duplicate *SPL* gene pair, the Ka/Ks ratio was calculated using MEGA 5.0 software to study divergence times. The Ka/Ks values in the pecan genome were distributed between 0.2–0.5, while the Ka/Ks ratios between the *Arabidopsis* and pecan genomes ranged from 0.1–0.4 (Fig. 5). This indicated that the *SPL* genes had a strong purifying selection between the pecan and *Arabidopsis* genomes.

### Analysis of cis-acting elements in the promoter regions of *CiSPL* genes

**Figure 6:**
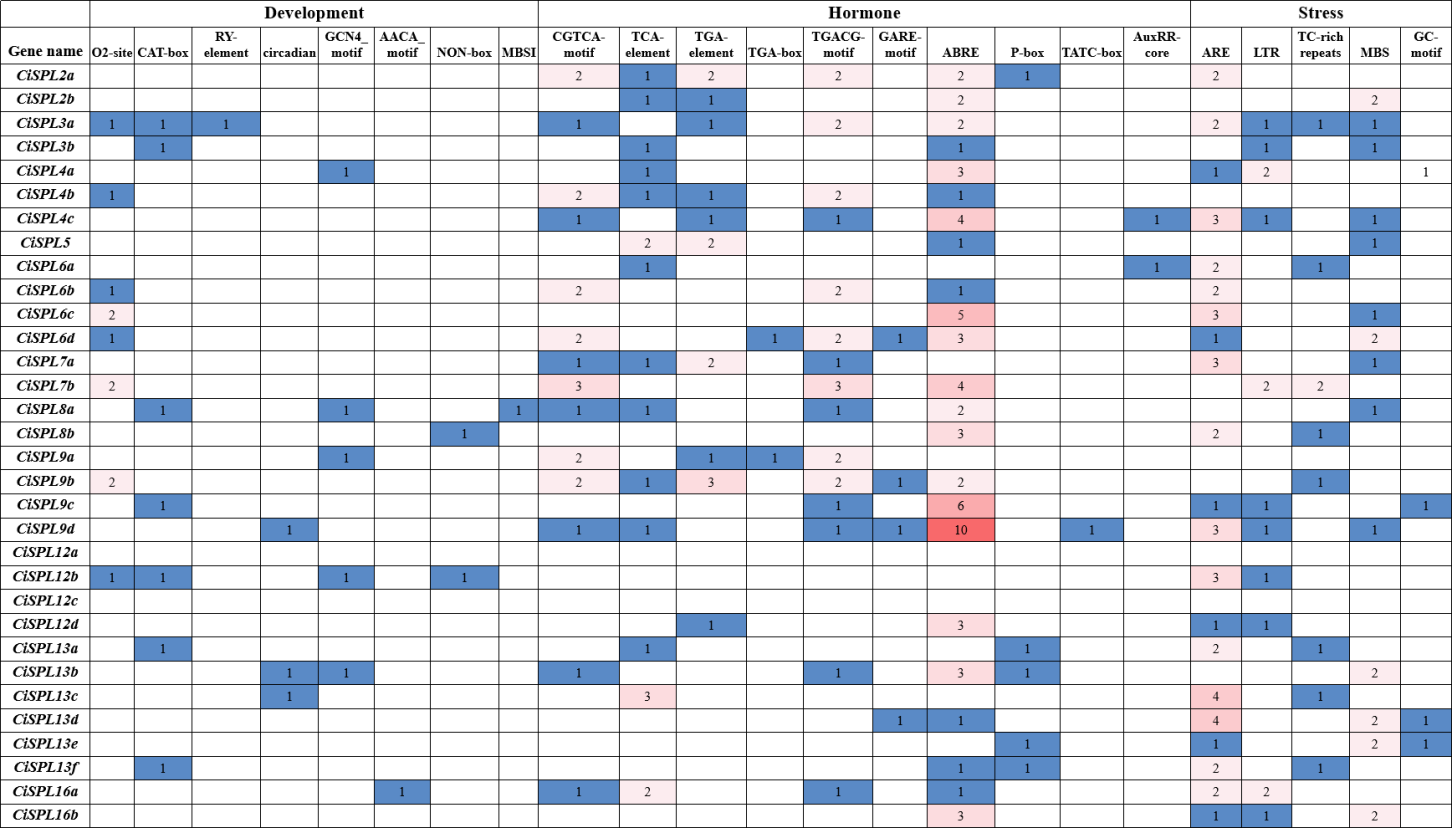
Analysis of cis-regulatory elements in promoter regions of *CiSPL* genes in pecan. The number of cis-acting elements in each *SPL* gene promoter region (1.5 kb upstream of the translation start site). Based on the functional annotation, the cis-acting elements were divided into three categories: development, hormone responsive, and stresses-related cis-acting elements.

We predicted and analyzed the cis-acting elements in the 1,500 bp promoter sequences of the *CiSPL* genes in order to understand the gene regulation mechanism. Except for the basic CAAT and TATA boxes, most of the other cis-acting elements mainly included stress response, hormone response, growth, and development, which were related to different life activities (Fig. 6). There were two cis-acting elements (GCN4_motif and AACA_motif) involved in endosperm expression and located in the promoter region of five and one *CiSPL* genes, respectively. The seed-specific regulation element (RY-element) was found only in the promoter region of *CiSPL5*. Nine meristem expression regulatory (NON-box and CAT-box) genes were identified in eight *CiSPL* promoters, and 11 zein metabolism regulation elements (O2 site) were also identified in eight *CiSPL* promoters. Additionally, circadian control (circadian) and flavonoid biosynthetic (MBSI) regulatory elements were located in the regions of three and one *CiSPL* promoters, respectively.

There were 46 MeJA-responsive elements (CGTCA motif and TGACG-motif), 19 auxin-responsive elements (TGA-element, TGA-box, and AuxRR-core), 10 gibberellin-responsive elements (GARE-motif, TATC-box, and P-box), 18 salicylic acid responsive elements (TCA-element), and 64 abscisic acid responsive elements (ABRE) located in the promoter regions of 15, 12, 9, 14, and 23 *CiSPLs*, respectively. There was the largest number of ABA-responsive elements, followed by MeJA-responsive elements, suggesting that *CiSPL* s may have a strong response to MeJA and ABA hormones, although this requires further experimental verification. The CiSPL transcription factor is an important adversity gene, and the number of hormone response elements in the promoters of *CiSPLs* varies widely. Among these, the *CiSPL25* gene has the largest number of hormone response elements in its promoter region (up to 15). The promoter region of the *CiSPL20* gene contains only one GA-related element, and no other types of hormone response elements. Different *CiSPL* s have different response patterns to different hormones.

There are five different types of stress-related elements in the promoter region of *CiSPL* genes. Among them, the most numerous are the regulatory elements related to anaerobic stress (ARE). Fourteen low temperature responsive elements (LTR) and 20 drought induction elements (MBS) were identified in 11 and 14 *CiSPL* genes, respectively. There are also stress-related response elements (TC_rich repeats).

### Expression profiles of *CiSPL* genes during flower and fruit development

**Figure 7:**
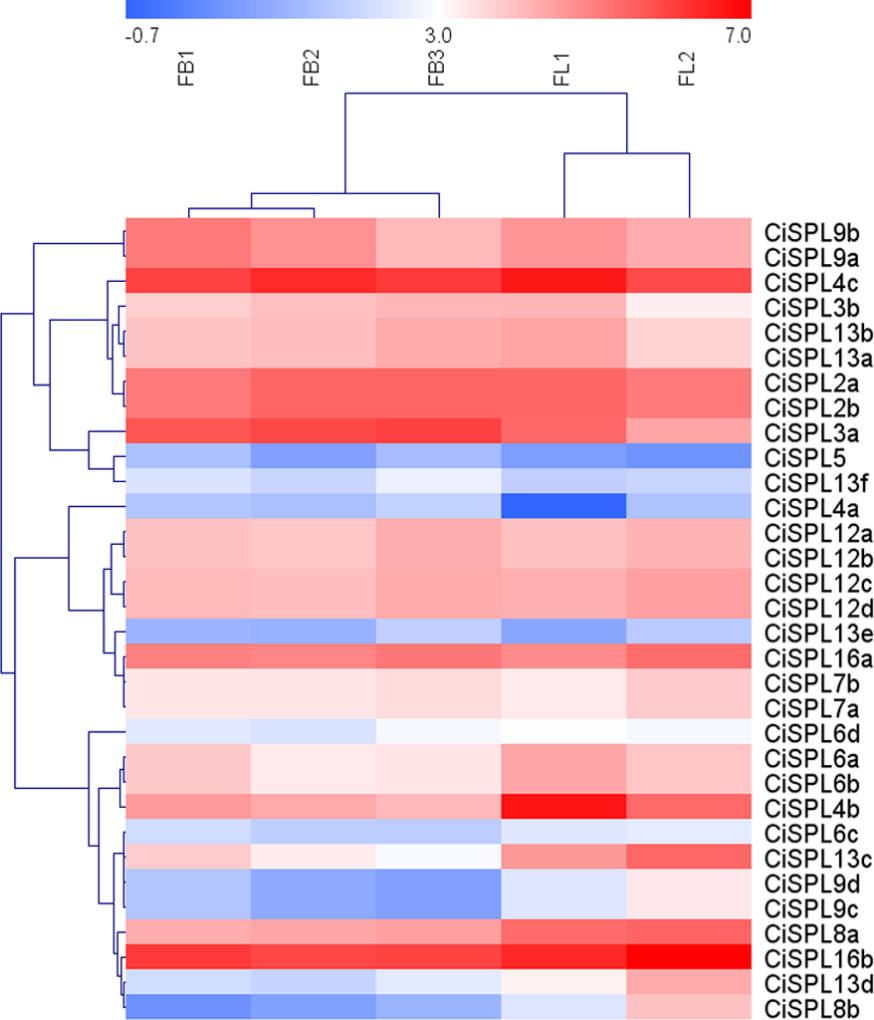
Expression profiles of *CiSPL.* genes during different stages of female flower development in pecan. Red and blue boxes indicate up-regulation and down-regulation of gene expression, respectively. The color key represents log2 expression values (FPKM) of the genes. FB1, initial stage of female flower bud differentiation; FB2, formation stage of female inflorescence; FB3, the formation stage of female flower involucre; FL1, initial flowering stage of female flowers; FL2, blooming period of female flowers.

**Figure 8:**
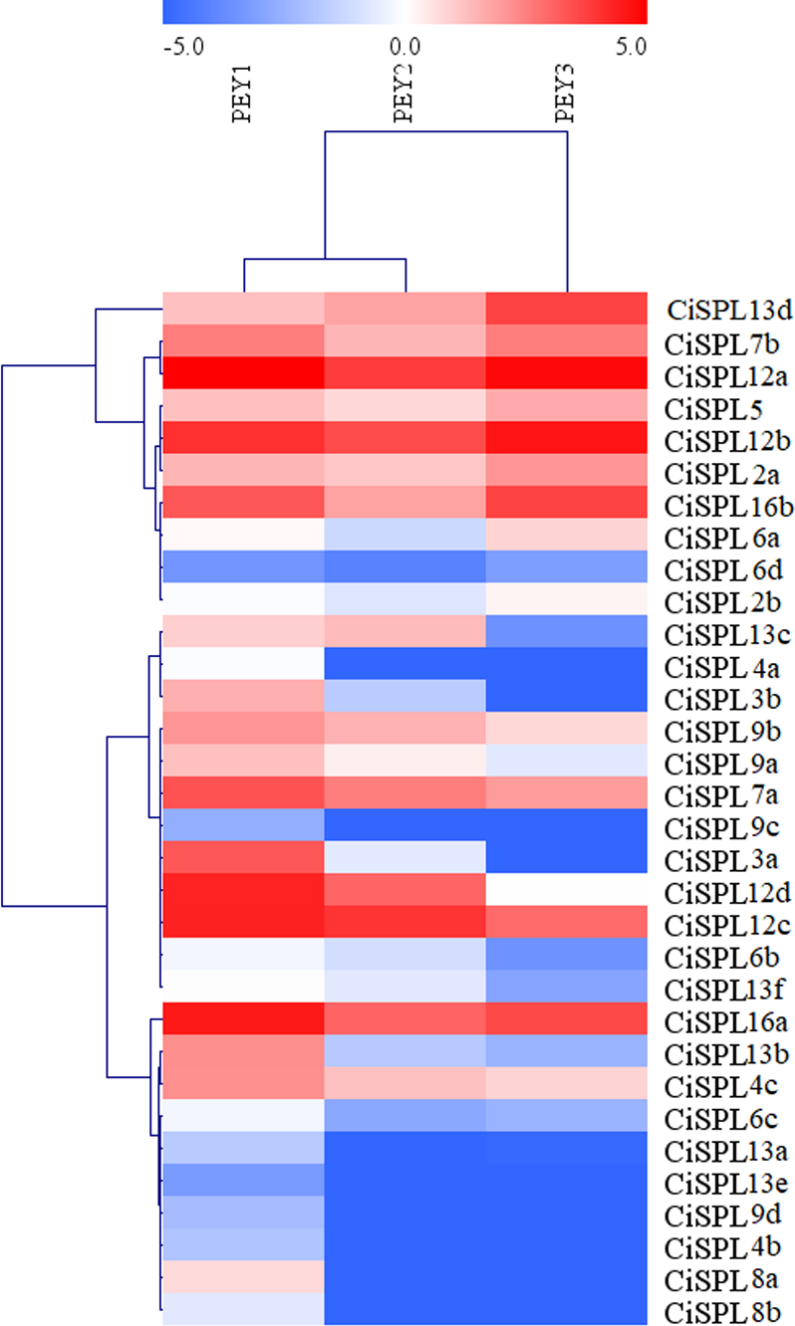
Expression profiles of *CiSPL* genes during different stages of fruit development in pecan. Red and blue boxes indicate up-regulation and down-regulation of gene expression, respectively. The color key represents log2 expression values (FPKM) of the genes. The relative expression was calculated by Log2(FPKM). PEY1, the early stage of cotyledon development; PEY2, the fully developed stage of cotyledon development; PEY3, the fully matured stage of the embryos.

In order to understand the function of *CiSPL* genes, we analyzed the expression patterns of identified *CiSPLs* using the transcriptome data of female flower development (Table S3). Figure 7 shows that two-thirds of *CiSPL* genes were expressed during female flower development.*CiSPL4c* and *CiSPL16b* showed a high expression level during the whole flower development stage (FB1 to FL2). Four *CiSPL* genes (*CiSPL8a*, *CiSPL8b*, *CiSPL9c*, and *CiSPL9d*) were only expressed during the FL2 stage. The *CiSPL4b* gene was particularly expressed in the FL1 stage. *CiSPLs* play different roles in different stages of female flower development, and there is differentiation of gene function. We also analyzed the expression of *CiSPL* genes across three stages of pecan fruit development (Fig. 8, Table S4). Surprisingly, about half of the *CiSPL* genes were not expressed during fruit development. *CiSPL12a*, *CiSPL12b*, and *CiSPL16a* genes were highly expressed throughout fruit development. *CiSPL3a* and *CiSPL12d* were mainly induced and expressed during the early stages of fruit development. The specific functions of these *CiSPL* genes need to be further verified in future experiments.

### Expression analysis of *CiSPL* genes under drought and NaCl stress treatment

**Figure 9:**
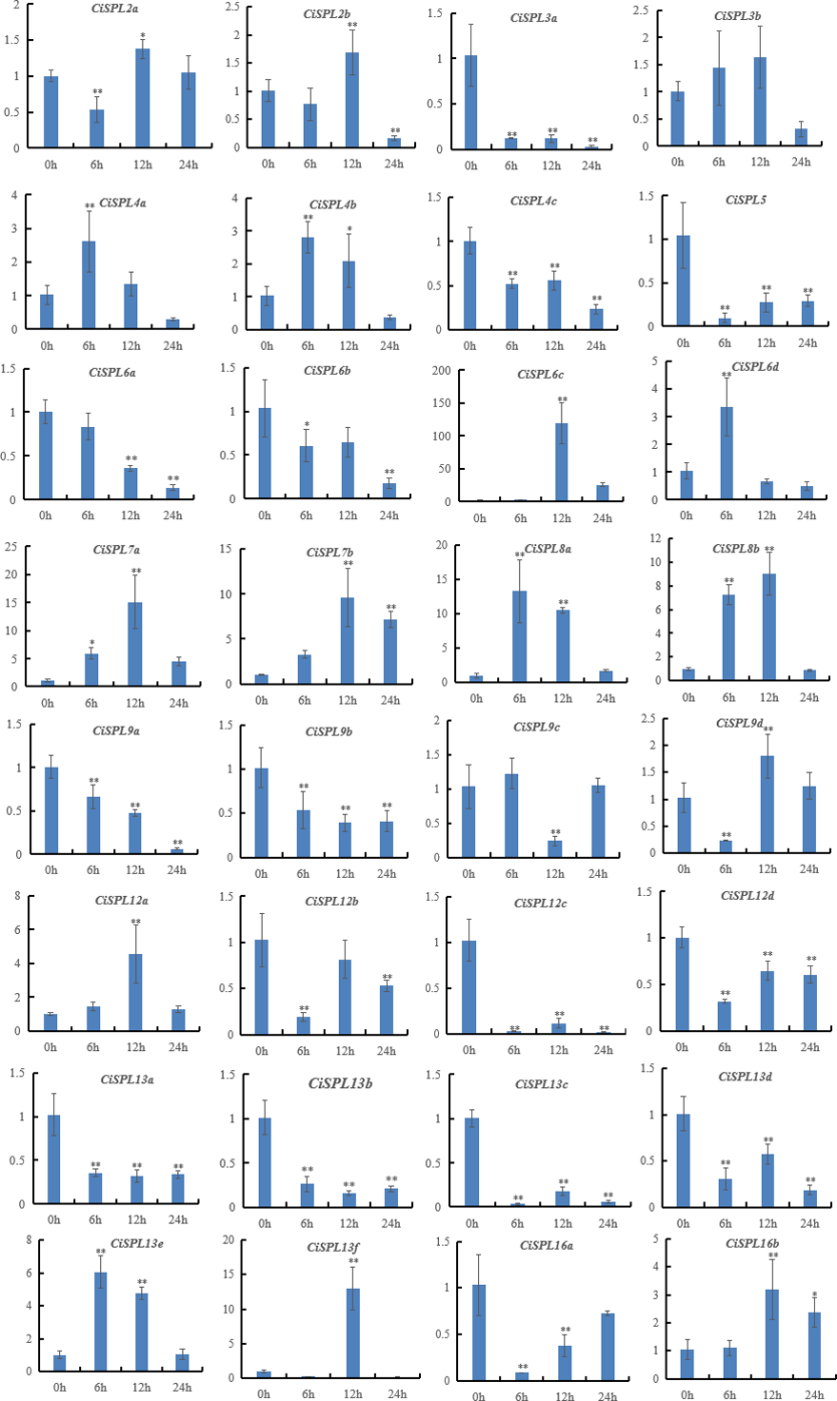
Expression profiles of 32 *CiSPL* genes under drought stress using qRT-PCR. The relative expression levels of *CiSPL* genes were normalized with respect to the reference gene *actin* under drought stress treatment. Error bars represent the standard deviations (SD). Asterisks on top of the bars indicate statistically significant differences between stress treatment and the control (^∗^0.01 < *P* < 0.05; ^∗∗^*p* < 0.01). *X*-axes show time courses of drought stress treatments for each gene. *Y*-axes indicate the scale of the relative expression levels.

In order to explore the response of *CiSPLs* to drought stress, the expression profiles of 32 *CiSPLs* were examined by qRT-PCR (Table S6). Figure 9 shows that nearly half of the *CiSPLs* were found to show a drought stress response. In general, shorter genes were expressed more quickly. Four of the shorter *CiSPLs* (*CiSPL4a*, *CiSPL4b*, *CiSPL6d*, *CiSPL8a*, and *CiSPL13e*) were induced rapidly and reached their peak at 6 h. *CiSPL7a*, *-7b*, *-12a*, *-13f*, and *-16b* expression reached their peak 12 h after drought stress. *CiSPL6c* was the most responsive gene, with an expression 100 times greater than that of control. Thirteen *CiSPLs* (*CiSPL-3a*, -*4c*, -*5*, -*6b*, -*9a*, -*9b*, *12c*, -*12d*, -*13a*, -*13b*, -*13c*, -*13d*, and -*16a*) were not induced or down-regulated during the whole process. Generally, *CiSPLs* play an important role in drought stress response.

**Figure 10:**
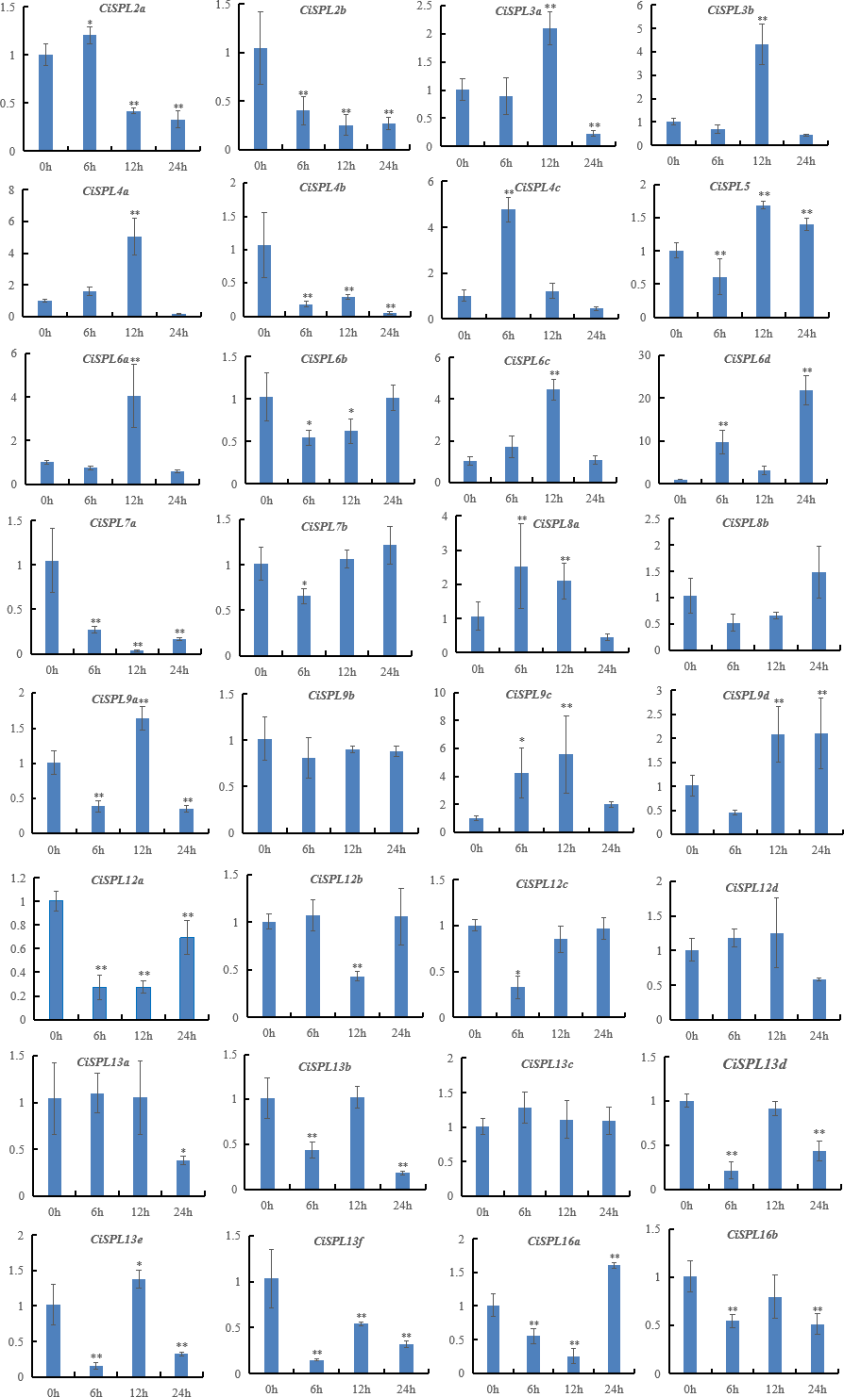
Expression patterns of 32 *CiSPL* genes under salt stress using qRT-PCR. Samples were collected 0, 6, 12, and 24 h after treatment, and the relative expression levels were analyzed. *X*-axes represent time points after salt treatment. *Y*-axes represent relative gene expression values normalized to reference gene *actin*. Error bars indicate the standard deviations (SD). Asterisks on top of the bars indicate statistically significant differences between the stress treatment and the control (^∗^0.01 < *P* < 0.05; ^∗∗^*p* < 0.01).

Under the NaCl treatment, nearly half of the 32 *SPL* genes were induced (Fig. 10, Table S7). However, six *CiSPLs* (*CiSPL2b*, *-4b*, *-7a*, *12a*, *-13f*, and *-16b*) were down-regulated during the whole stress treatment process. *CiSPL4c* was significantly expressed at 6 h. The transcription level of six genes (*CiSPL3a*, *-3b*, *-4a*, *-6a*, *-6c*, and *-9c*) reached the maximum at 12 h. *CiSPL8a* and *CiSPL13e* were slightly induced at 12 h. *CiSPL6d* gene expression reached its peak at 24 h (nearly 20-fold over the control). *CiSPL9d* was induced at 12 h and 24 h, and the expression level was nearly the same.

## Discussion

It has been established that the SPL gene family of plant specific transcription factors plays important roles in plant growth and development, as well as abiotic and biotic stress. Previous research on SPL transcription factors mainly focused on model plants. In this study, the function of *SPLs* was identified and investigated in a woody species, *Carya illinoinensis*. The whole genome identification of SPL transcription factors has been reported in several plant species with varying results (Table S5). The numbers were relatively conservative among Arabidopsis (17 members), grapevine (18 members), jujube (18 members), chestnut (18 members), rice (19 members), and strawberry (19 members). Recent studies also identified 30 *SPL* genes in hickory (*Carya cathayensis*), 30 *SPLs* in pecan (*Carya illinoensis*), and 37 *SPLs* in *Juglans regia* (Wu et al., 2019). However, a different study identified 48 SBP-box genes from *Juglans regia* (Zhou et al., 2020). In this study, 32 *SPL* genes were identified in the pecan genome, which was two more *SPL* genes than what was found in previous studies. We also identified the SPL gene family of pecan from the newly released genome (https://phytozome-next.jgi.doe.gov/info/CillinoinensisPawnee_v1_1) (Lovell et al., 2021). The number of SPL gene family members was found to be the same in the two versions of the pecan genome (Table S8). This indicates that the number of *SPL* genes is similar to that of different genera of *Juglandaceae,* apple (27 members), and poplar (28 members).

Using the phylogenetic tree analysis and protein sequence multiple alignment, the SPL proteins from pecan, *Arabidopsis*, rice, and poplar were divided into eight subgroups (I–VIII). Each subgroup had an unequal number of genes from pecan, *Arabidopsis*, and rice, while the numbers of pecan and poplar genes in each subgroup was similar. Phylogenetic analysis showed that the *CiSPL* genes were closely associated with *PtSPL* genes, which is consistent with the fact that pecan and poplar are woody plants. *CiSPL* genes’ diverse gene structures and protein motifs can help better understand their different roles in development and growth. Gene structure analysis showed that subgroup II *CiSPL* genes had the largest number of introns (nine), while other subgroups contained only two to three introns. Interestingly, two members (*CiSPL4c* and *CiSPL9d*) had no introns. Previous studies have shown that complete intron loss contributes to a new gene generation (Volokita, Rosilio-Brami & Rivkin, 2011). Intron variation in *CiSPLs* may be caused by two mechanisms: intron loss and intron gain. *SPL* genes has been amplified in a large number of plant genomes. The main amplification mechanisms are tandem and/or segmental duplication (Cannon et al., 2004). In our analysis, the segmentally duplicated gene pairs of *CiSPLs* accounted for over 50% of the entire SPL gene family in pecan (Fig. 4), but no tandem duplications were found. This result supported the interpretation that segmental duplication is the main reason for the amplification of SPL gene family members in pecan. To explore the macro evolution model in pecan, the Ka/Ks ratios for the segmentally duplicated gene pairs were estimated (Lan et al., 2019). Coincidentally, the peak values of Ka/Ks ratios for the *Ci*-*Ci* and *Ci*-*At* gene pairs were both between 0.3 and 0.4, implying a strong selection constraint and purifying selection in the *CiSPL* genes.

Pecan has a long vegetative growth period, late flowering and fruiting, and poor economic benefit, which have restricted its industrial development (Randall et al., 2015; Zhang, Peng & Li, 2015). Recent research on pecan flower bud differentiation, domestically and abroad, has mainly focused on phenological observation, external morphology, and internal anatomical structure. In our study, most *CiSPL* genes displayed different expression levels at different floral stages, implying that *CiSPL* genes mainly play roles in pecan flower development. Similarly to walnut, most of the *JrSBP* genes play an important role in flower induction (Zhou et al., 2020). In *Arabidopsis, AtSPL3*, *SPL4,* and *SPL5* primarily promoted floral induction and/or floral meristem identity (Yamaguchi et al., 2009; Cardon et al., 1997; Wang, Czech & Weigel, 2009). Phylogenetic analysis showed that *AtSPL3* was highly orthologous to *CiSPL3a* and *CiSPL3b*, while *AtSPL4* and *AtSPL5* shared close relationships with *CiSPL4a*, *CiSPL4b*, *CiSPL4c*, *and CiSPL5.* Four (*CiSPL3a*, *CiSPL3b*, *CiSPL4b*, and *CiSPL4c*) of the six *CiSPL* genes were expressed during the flower development process, indicating that these genes may be involved in the development of the flower. In *Arabidopsis*, *AtSPL2*, *AtSPL10*, and *AtSPL11* jointly regulated the development of lateral organs during the reproductive stage, and were also involved in regulating the morphogenesis of stem leaves and flowers (Shikata et al., 2009). *AtSPL2*, *AtSPL10,* and *AtSPL11* were highly orthologous to *CiSPL2a* and *CiSPL2b*, which were mainly up-regulated during the transformation from stem apical meristem (SAM) to floral meristem (undifferentiated, early differentiated, and inflorescence forming stages). Some *CiSPL* genes appeared to show similar expression patterns during specific pecan stages, which may reflect their participation in a common developmental process. For example, some genes in subgroup I (*CiSPL13c*/*13d*) and III (*CiSPL8a*/*8b*) had high expression during the flowering stage, while several genes in subgroup V (*CiSPL3a*/*3b*), were highly expressed in the flower buds. Therefore, we concluded that gene function differentiation existed in the same subfamily.

Previous studies focused on the role of *SPL* genes in development, but the function of *SPL* genes under stress has been seldom studied. In our study, the *CiSPL* genes showed diverse expression patterns under drought and salt stresses. qRT-PCR analysis showed that six SPL genes (*CiSPL4a*, *CiSPL6c*, *CiSPL6d*, *CiSPL8a*, *CiSPL9d*, and *CiSPL13e*) were up-regulated under the two stress treatments, confirming that these *CiSPL* s may actively participate in regulating stress responses. Gene expression regulation is mainly carried out at the transcription level, which is coordinated by a variety of cis-acting elements and trans-acting factors. TC-rich repeats and MBS were found in 21 *CiSPL* promoter regions. Analysis of *CiSPL* expression levels showed that these *CiSPLs* were strongly affected by at least one stress. Among the 11 genes that were mainly induced by drought stress, eight genes had promoter regions that contained MBS cis-elements. Some *CiSPL* genes were not induced under either drought or NaCl stress (*CiSPL13a*, *CiSPL13b*, *CiSPL13c*, and *CiSPL13d*, belonging to subgroup VII), and showed low gene expression under the two stresses.

In our study, there were 16 collinear gene pairs between Arabidopsis and pecan. Most of these orthologs were one-to-one relationships, although some orthologs showed one-to-two relationships. Among the two gene pairs (*CiSPL6a*/*6b*-*AtSPL6, CiSPL16a*/*16b*-*AtSPL16)*, *CiSPL6a* and *CiSPL6b* expression showed differences under salt stress and during fruit development, while *CiSPL16a* and *CiSPL16b* expression differed under drought and salt stress. We inferred that these duplicated gene pairs experienced functional divergence, and one of them acquired a new function: ‘neofunctionalization’. The other gene pairs presented the same expression pattern, which showed that most collinear genes had conservative functions.

## Conclusion

The *CiSPLs* were characterized using detailed analyses of their phylogenetic relationships, gene structure, conserved motifs, duplication events, and expression profiles. A total of 32 *CiSPL* genes were identified from the pecan genome in this study. The *CiSPLs* were divided into eight groups based on their phylogenetic analysis and homology with Arabidopsis. There were similar gene structures and motifs in the same subgroups. Cis-element analysis showed that *CiSPLs* were regulated by plant hormones and various stresses. The *CiSPLs* played an important role in the flower development process. *CiSPL* genes also showed distinct spatiotemporal expression patterns in response to drought and salt treatment. In general, our data increased our understanding of the evolutionary relationships and biological functions of *SPL* genes in pecan. Ultimately, the results of this study lay a foundation for revealing the further functional characterization of the SPL gene family.

## Supplemental Information

10.7717/peerj.12490/supp-1 Supplemental Information 1 Multiple alignments of the conserved SBP-domains for the pecan SPL proteins The SBP conserved domain contains two zinc finger structures and one NLS structure.

10.7717/peerj.12490/supp-2 Supplemental Information 2 The specific primers of all *CiSPL* genes used in qRT-PCR analysis

10.7717/peerj.12490/supp-3 Supplemental Information 3 Protein sequences and lengths of the major motifs identified by MEME in the putative CiSPL proteins

10.7717/peerj.12490/supp-4 Supplemental Information 4 Transcriptome expression data (FPKM) for the 32 *SPL* genes in pecan during female flower development

10.7717/peerj.12490/supp-5 Supplemental Information 5 Transcriptome expression data (FPKM) of the pecan *CiSPL* genes during fruit development

10.7717/peerj.12490/supp-6 Supplemental Information 6 Gene numbers of different subgroups of SPL family in four species

10.7717/peerj.12490/supp-7 Supplemental Information 7 The raw data for RT-qPCR analysis of 32 *CiSPLs* under drought stress

10.7717/peerj.12490/supp-8 Supplemental Information 8 The raw data for RT-qPCR analysis of 32 *CiSPLs* under salt stress

10.7717/peerj.12490/supp-9 Supplemental Information 9 The identification of *CiSPL* genes from two versions of pecan genome

## Additional Information and Declarations

### Data Availability

The following information was supplied regarding data availability: The raw measurements and data are available in the Supplementary Files.
