# Supplementary figures and images for: Characterization and expression analysis of the SPL gene family during floral development and abiotic stress in pecan (Carya illinoinensis)
Source: PeerJ. 2021 Dec 9;9:e12490. doi: 10.7717/peerj.12490 (PMC8667720; doi:10.7717/peerj.12490)

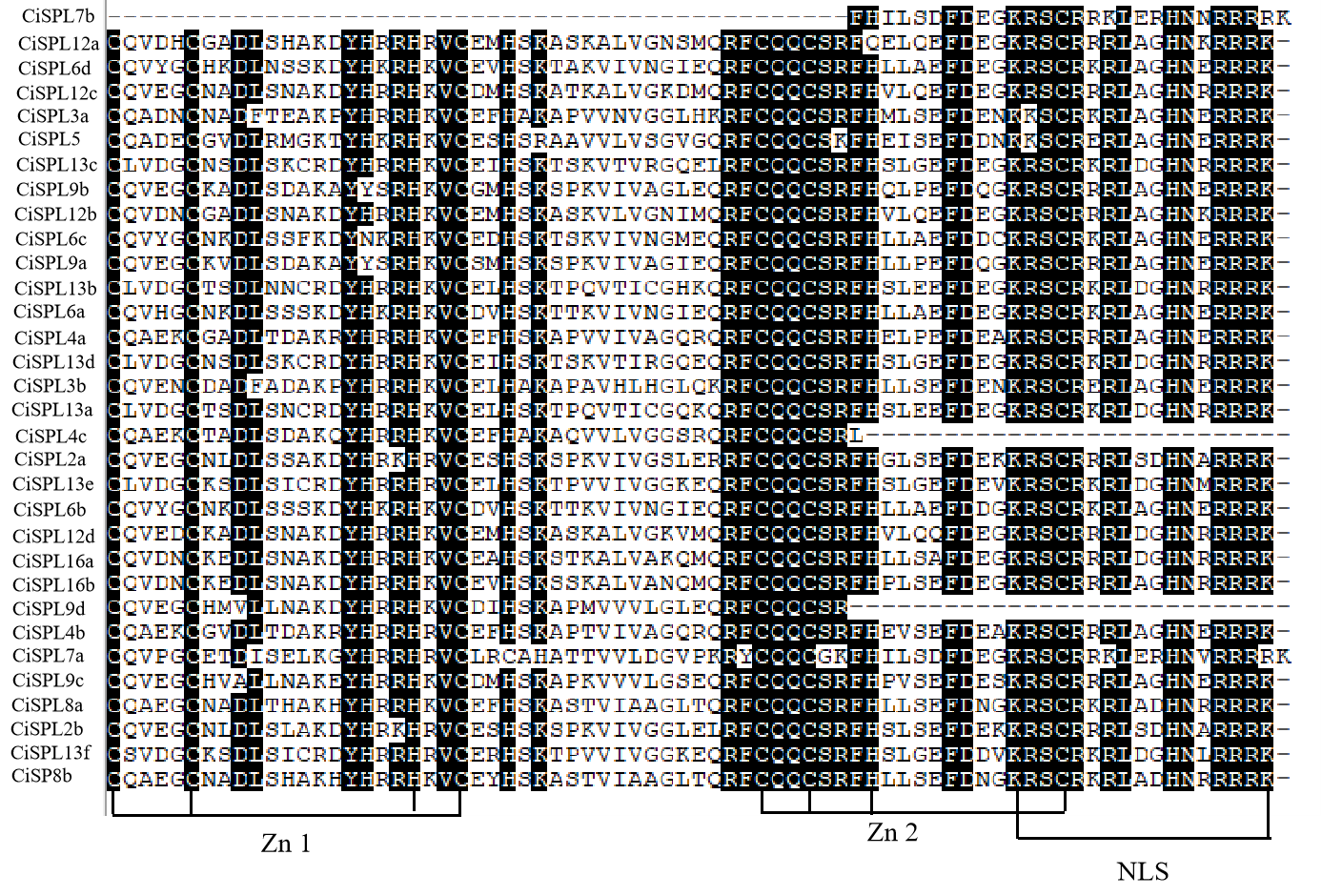

Supplement: Supplemental Information 1 — The SBP conserved domain contains two zinc finger structures and one NLS structure. [file peerj-09-12490-s001.png]
