# Supplementary material for: Characterization and expression analysis of the SPL gene family during floral development and abiotic stress in pecan (Carya illinoinensis)
Source: PeerJ. 2021 Dec 9;9:e12490. doi: 10.7717/peerj.12490 (PMC8667720; doi:10.7717/peerj.12490)
Supplement: Supplemental Information 3 [file peerj-09-12490-s003.docx]

Table S2. Protein sequences and lengths of the major motifs identified by MEME in the putative CiSPL proteins.


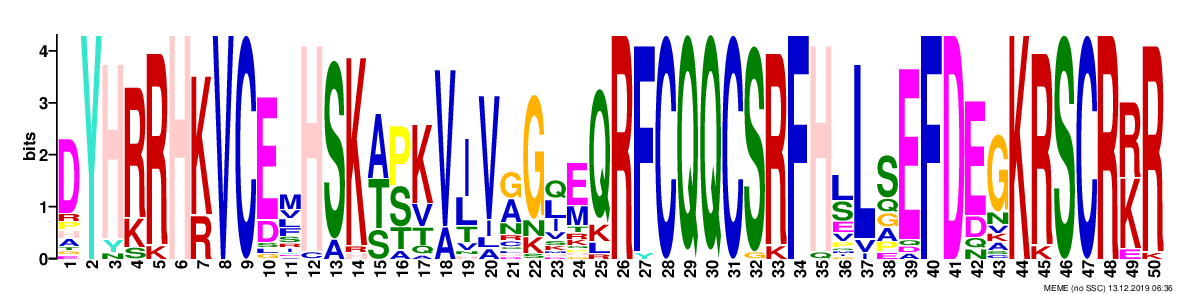


Motif 1


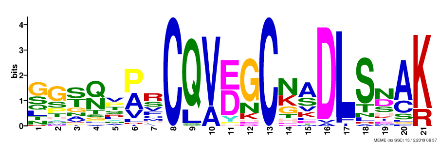


Motif 2


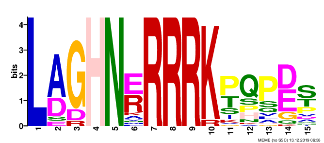


Motif 3


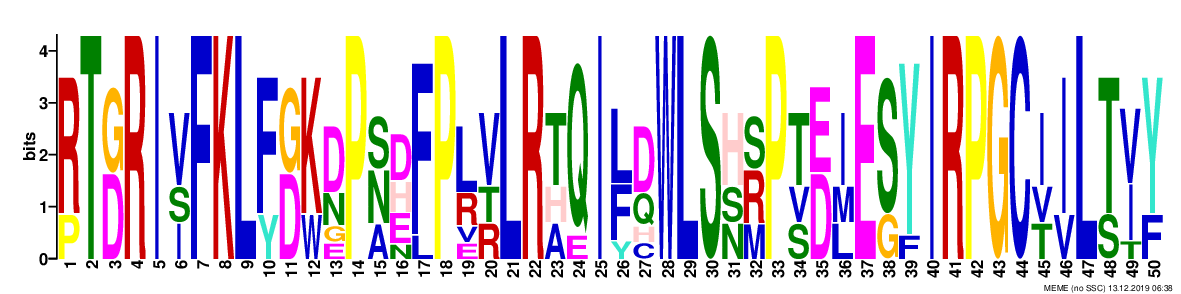


Motif 4


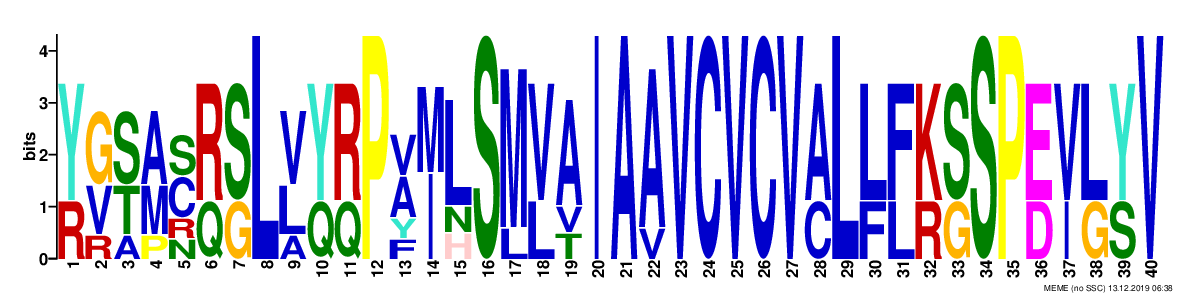


Motif 5


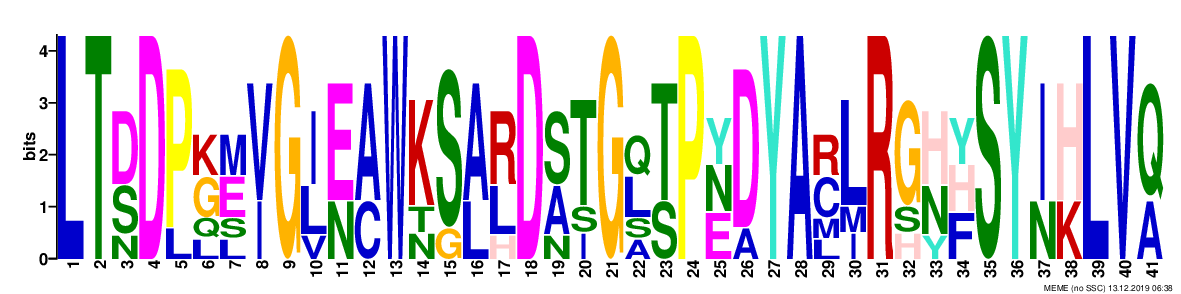


Motif 6


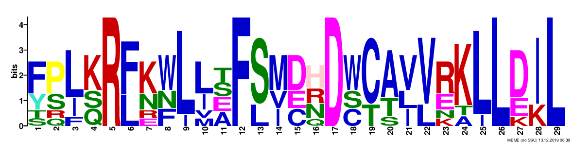


Motif 7


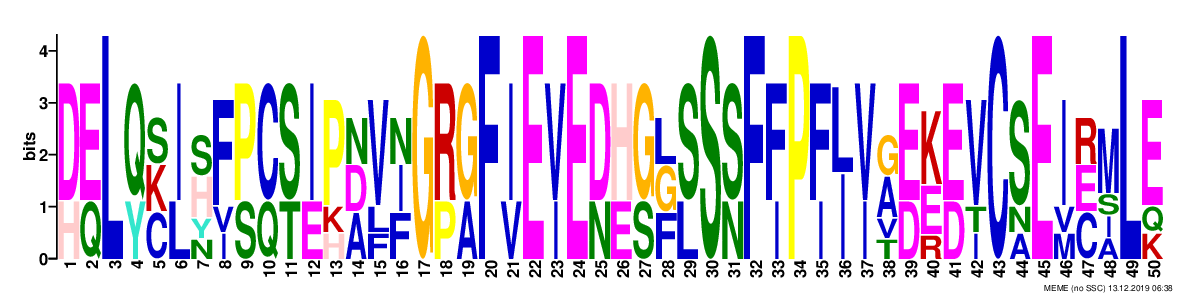


Motif 8


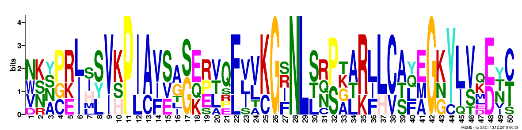


Motif 9


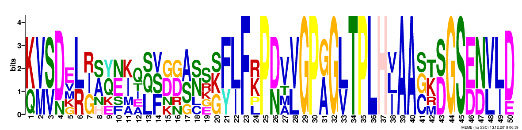


Motif 10


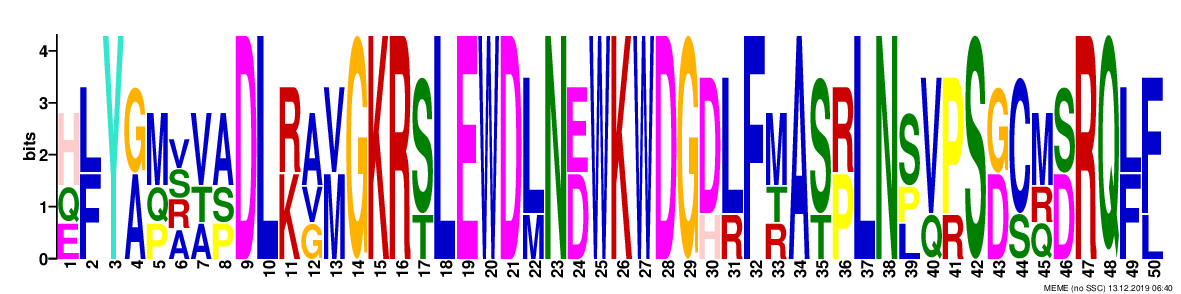


Motif 11


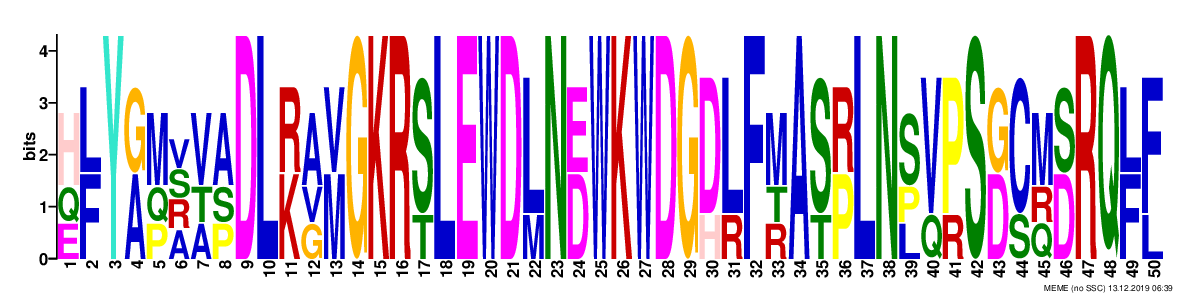


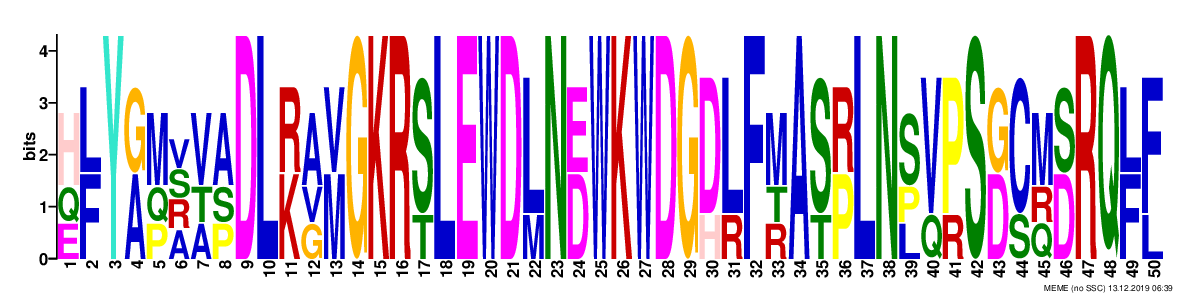


Motif 12


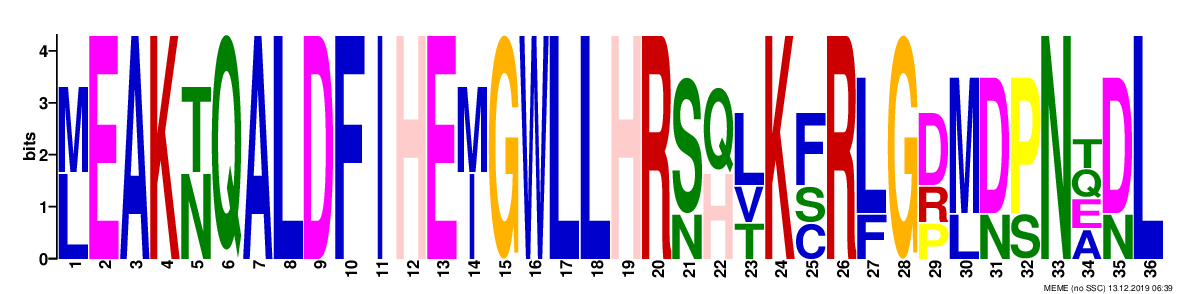


Motif 13


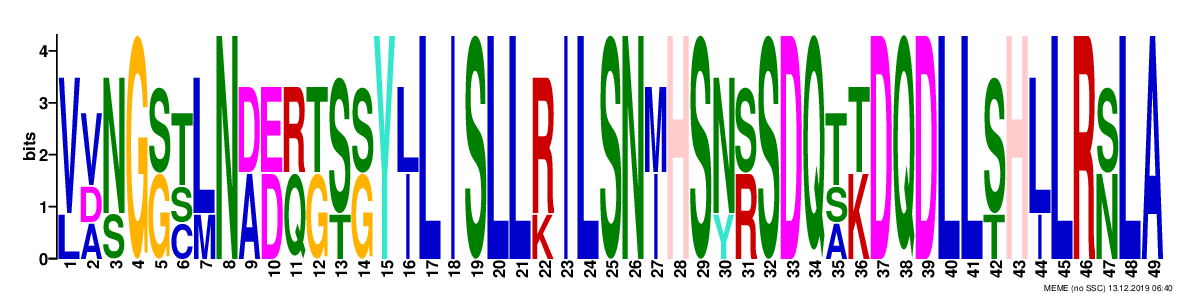


Motif 14


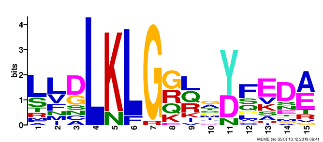


Motif 15


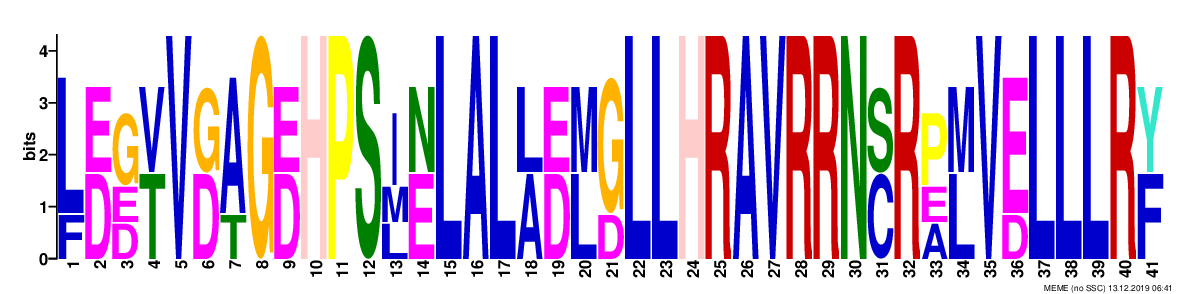


Motif 16


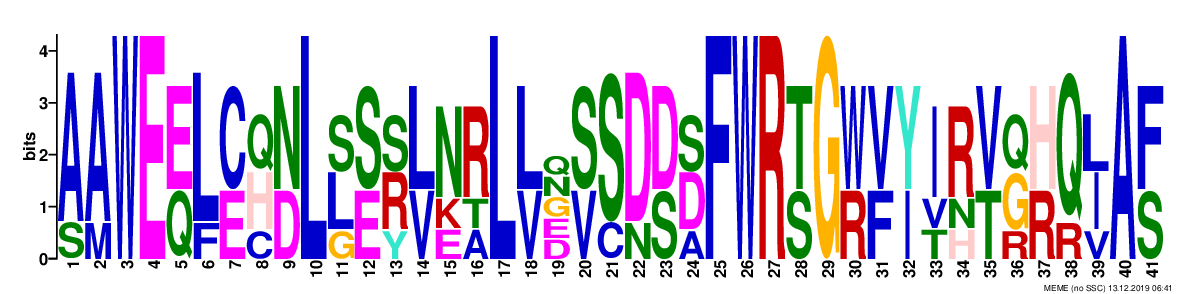


Motif 17


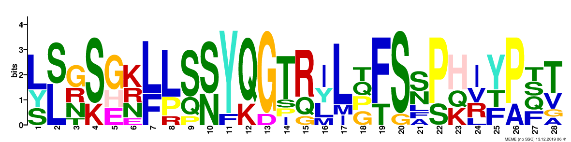


Motif 18


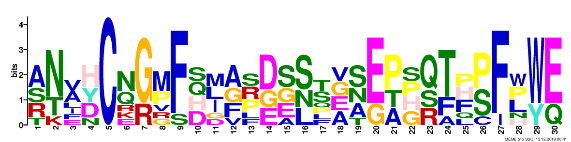


Motif 19


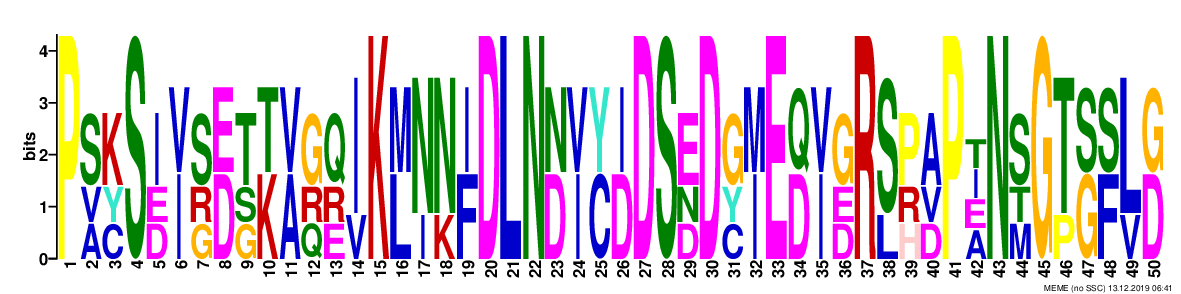


Motif 20
