# Supplementary material for: Characterization and expression analysis of the SPL gene family during floral development and abiotic stress in pecan (Carya illinoinensis)
Source: PeerJ. 2021 Dec 9;9:e12490. doi: 10.7717/peerj.12490 (PMC8667720; doi:10.7717/peerj.12490)
Supplement: Supplemental Information 6 [file peerj-09-12490-s006.docx]

Table S5 Gene numbers of different subgroups of SPL family in four species

| **Group** | **Pecan** | **Arabidopsis** | **Rice** | **Poplar** |
| --- | --- | --- | --- | --- |
| I | 2 | 1 | 1 | 2 |
| II | 6 | 4 | 2 | 6 |
| III | 2 | 1 | 3 | 2 |
| IV | 4 | 1 | 1 | 3 |
| V | 2 | 3 | 5 | 3 |
| VI | 6 | 3 | 1 | 5 |
| VII | 6 | 2 | 3 | 4 |
| VIII | 4 | 2 | 2 | 3 |
| Total | 32 | 17 | 18 | 28 |
